# Supplementary material for: Statistical evaluation of character support reveals the instability of higher-level dinosaur phylogeny
Source: Sci Rep. 2023 Jun 7;13:9273. doi: 10.1038/s41598-023-35784-3 (PMC10247814; doi:10.1038/s41598-023-35784-3)
Supplement: Supplementary file 1 — Supplementary Information. [file 41598_2023_35784_MOESM1_ESM.pdf]

Supplementary Information

Statistical evaluation of character support reveals the instability of higher-level dinosaur phylogeny

David Černý[1,*] & Ashley L. Simonoff[1,†]

[1]*Department of the Geophysical Sciences, University of Chicago, 5734 South Ellis Avenue, Chicago, IL 60637, USA*

[*]***Corresponding Author.*** *Email: david.cerny1@gmail.com*

[†]*Email: ashleysimonoff@gmail.com*

Metric	BEA	LEA
# of trees (n_{all})	100	100
# of plausible trees (n_{pl})	67	51
Avg. pairwise normalized RF distance, all trees ($\bar{d}_{\mathrm{all}}$)	0.3296	0.3952
Avg. pairwise normalize RF distance, plausible trees ($\bar{d}_{\mathrm{pl}}$)	0.3203	0.3618
# of unique topologies, all trees (n'_{all})	100	100
# of unique topologies, plausible trees (n'_{pl})	67	51

Supplementary Table 1: Metrics computed to evaluate the difficulty of estimating higher-level dinosaur phylogeny from the Baron et al. (BEA) and Langer et al. (LEA) datasets. RF distance = Robinson-Foulds distance.

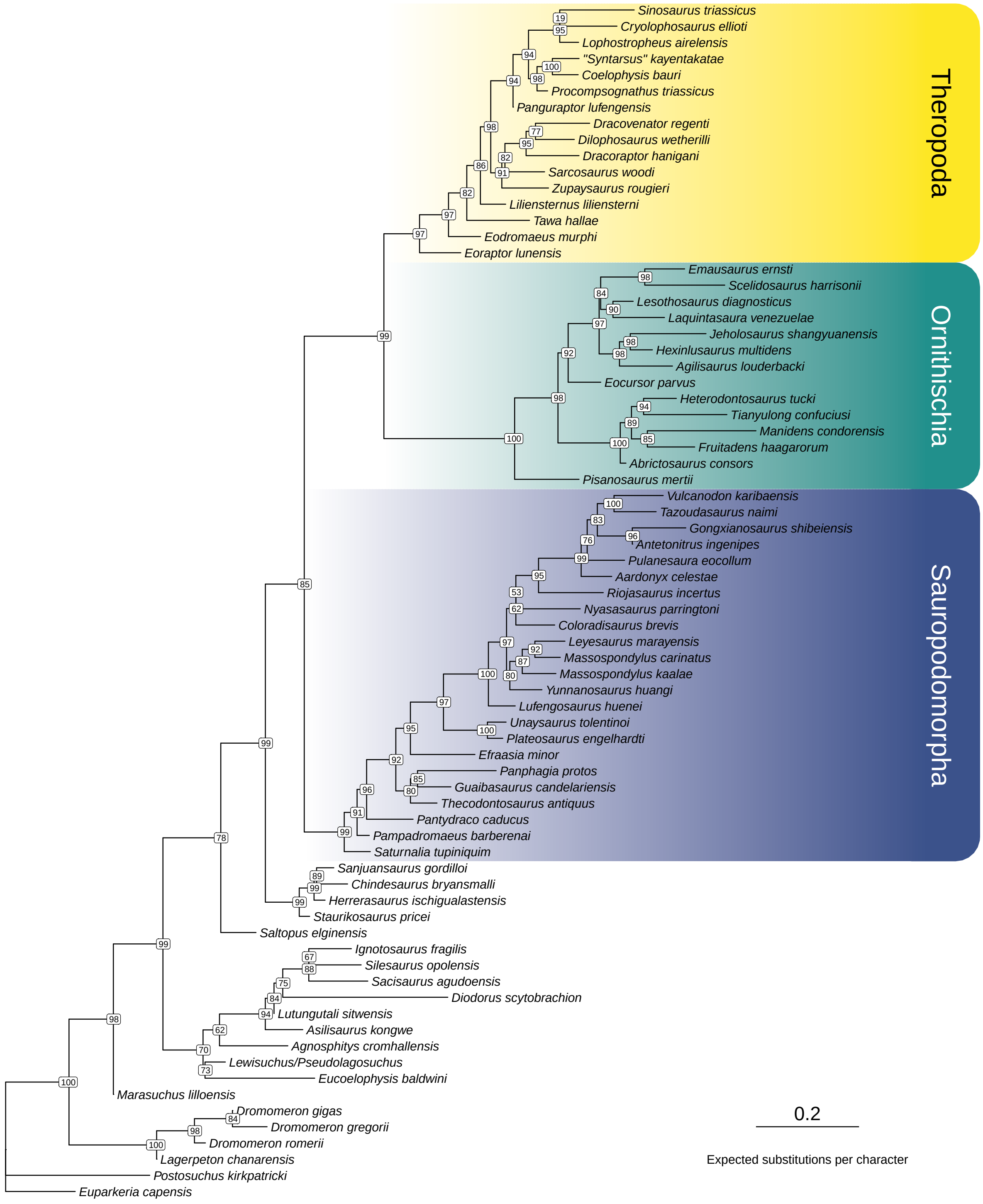

Supplementary Fig. 1: Unconstrained maximum likelihood tree inferred for the original BEA matrix ($\ln L = -6367.975$). Node labels denote ultrafast bootstrap values computed from 1000 replicates.

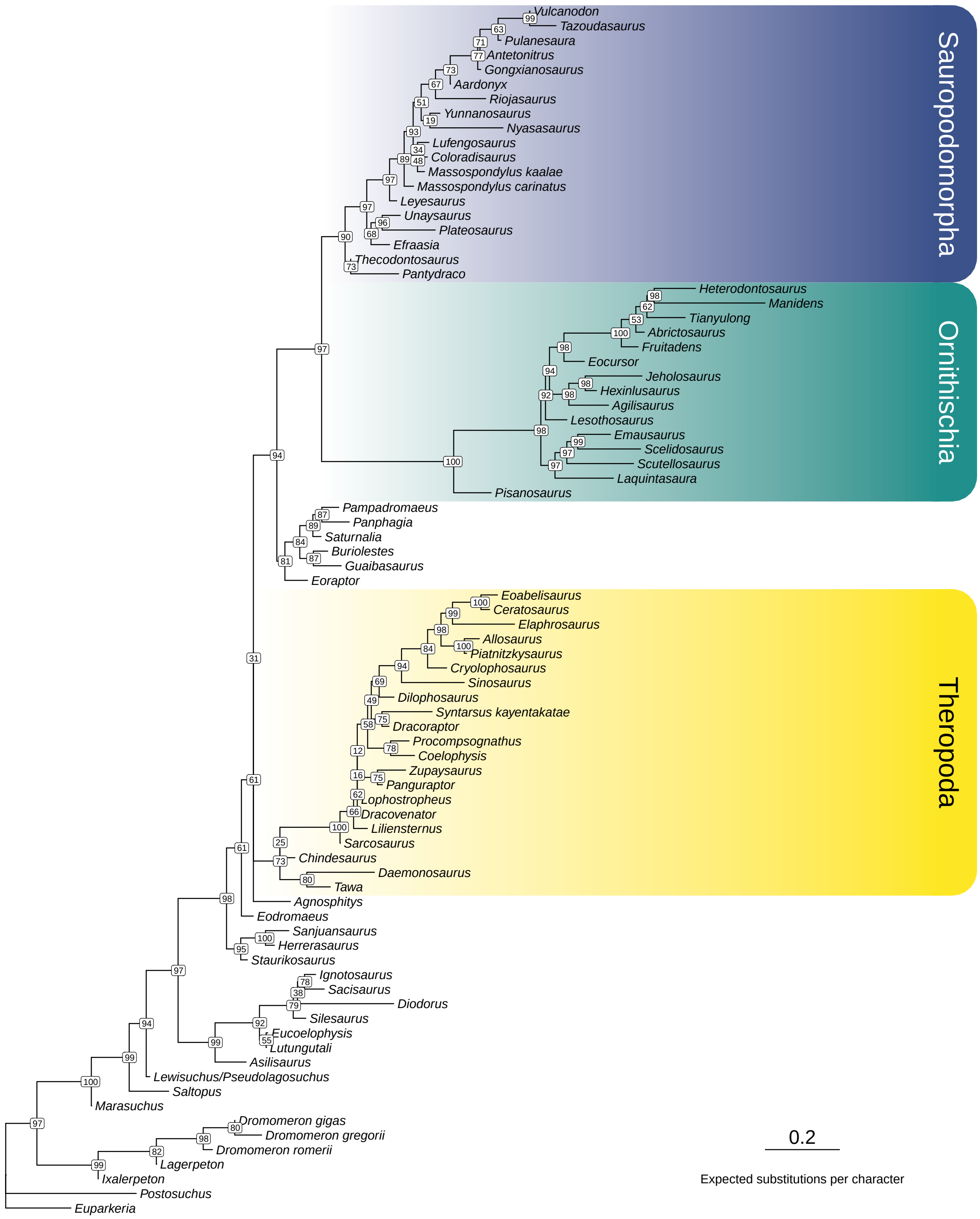

Supplementary Fig. 2: Unconstrained maximum likelihood tree inferred for the original LEA matrix ($\ln L = -7007.117$). Node labels denote ultrafast bootstrap values computed from 1000 replicates.

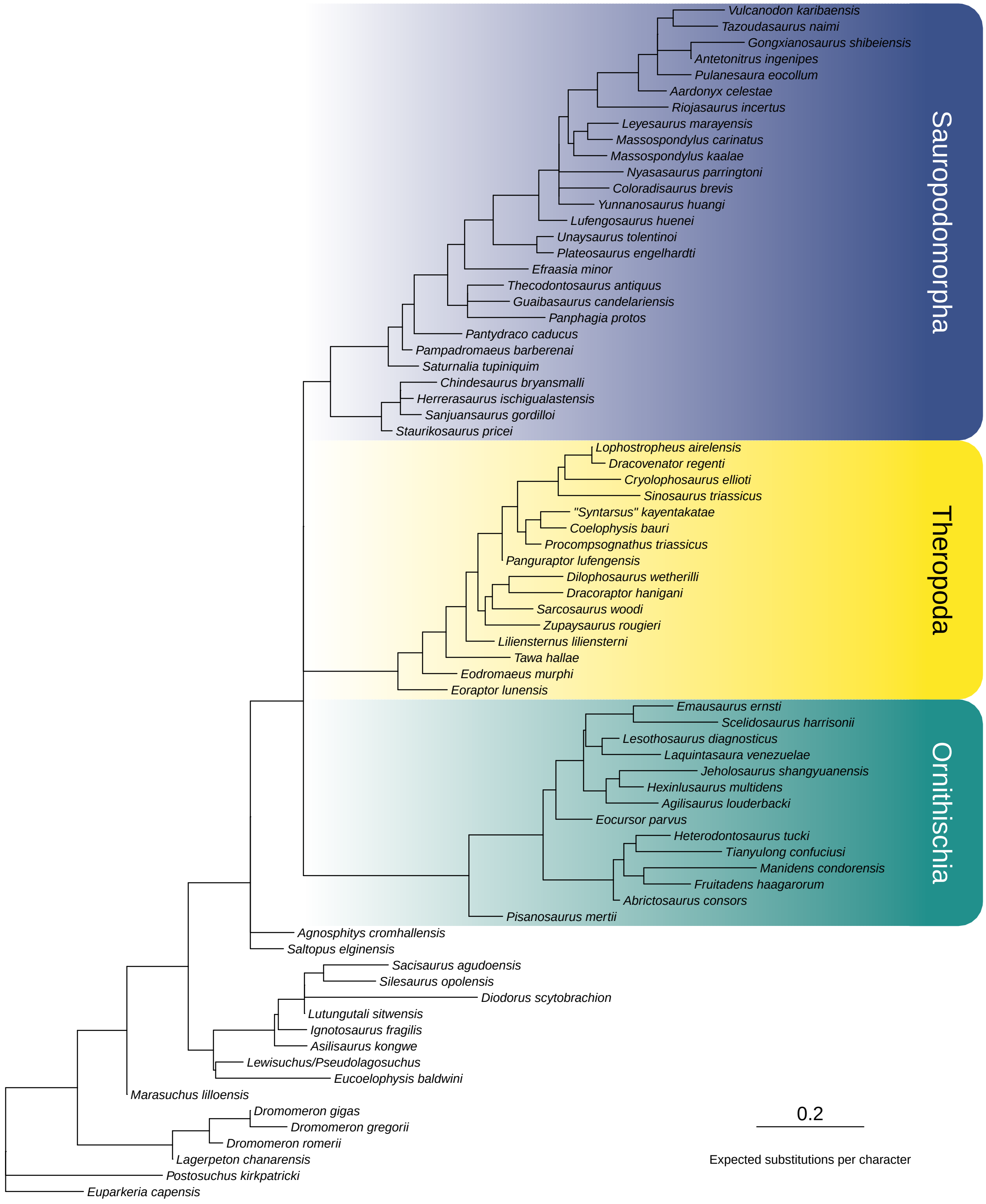

Supplementary Fig. 3: Maximum likelihood tree inferred for the original BEA matrix and constrained to recover Saurischia ($\ln L = -6376.854$).

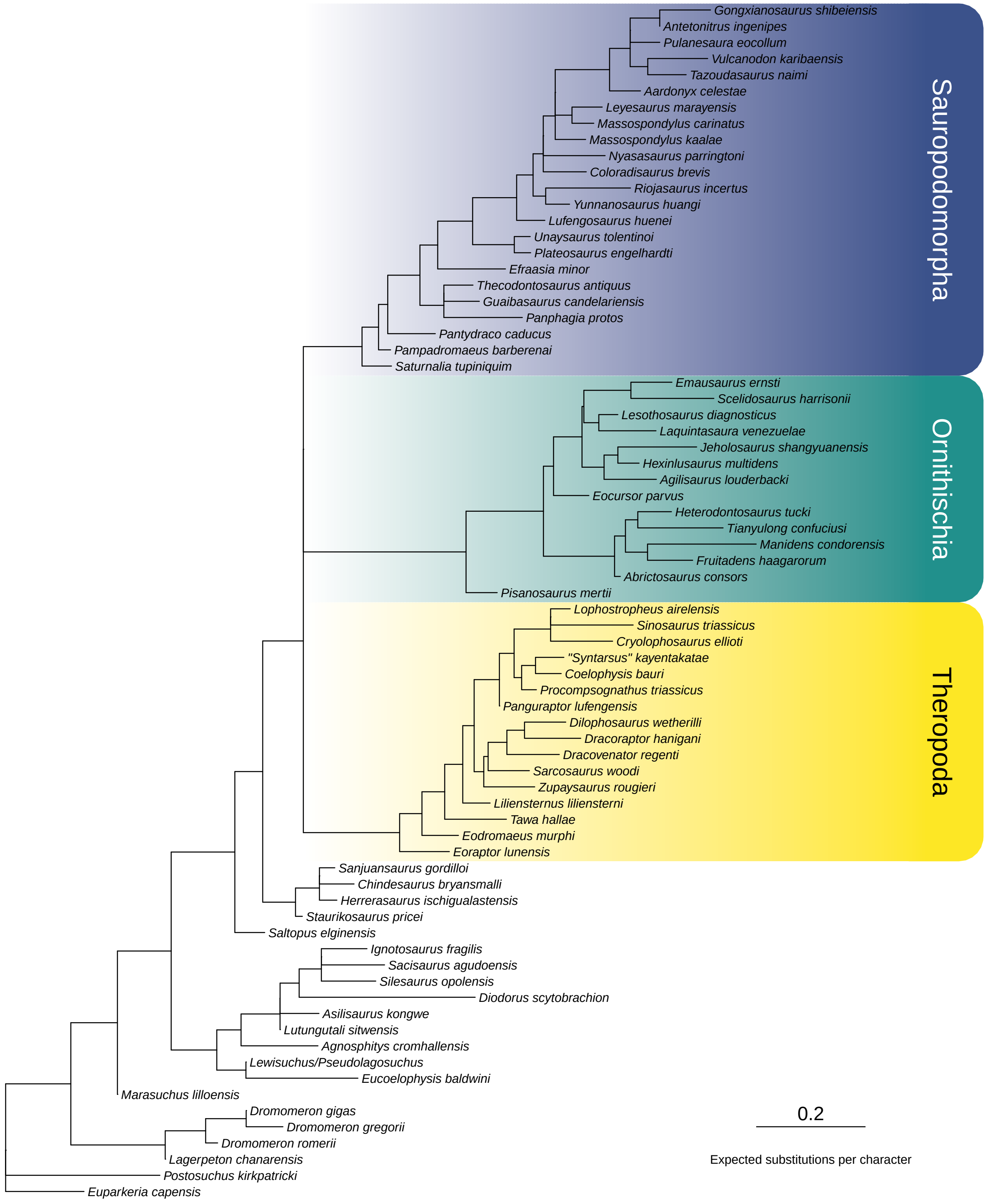

Supplementary Fig. 4: Maximum likelihood tree inferred for the original BEA matrix and constrained to recover Ornithischiformes ($\ln L = -6377.425$).

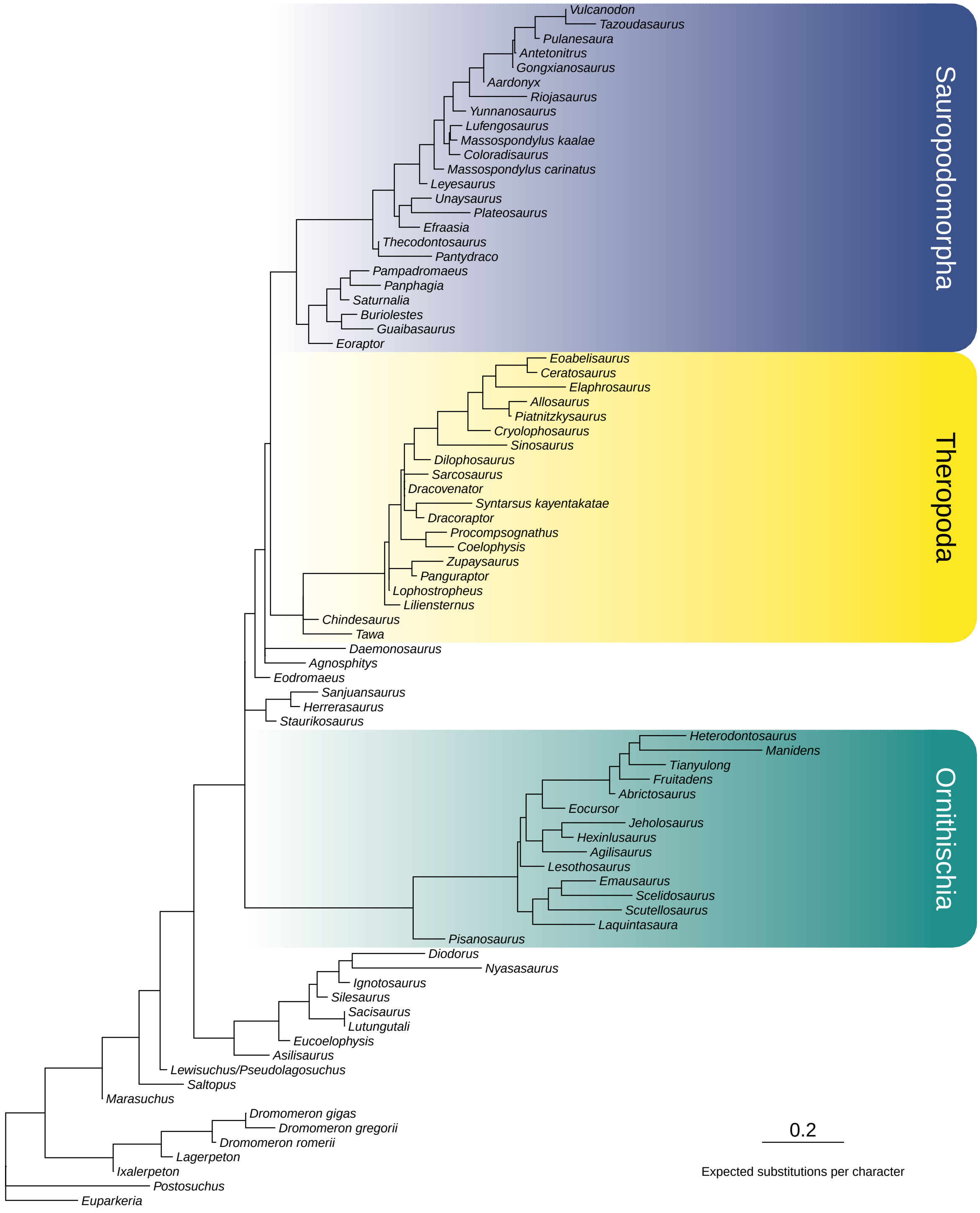

Supplementary Fig. 5: Maximum likelihood tree inferred for the original LEA matrix and constrained to recover Saurischia ($\ln L = -7017.246$).

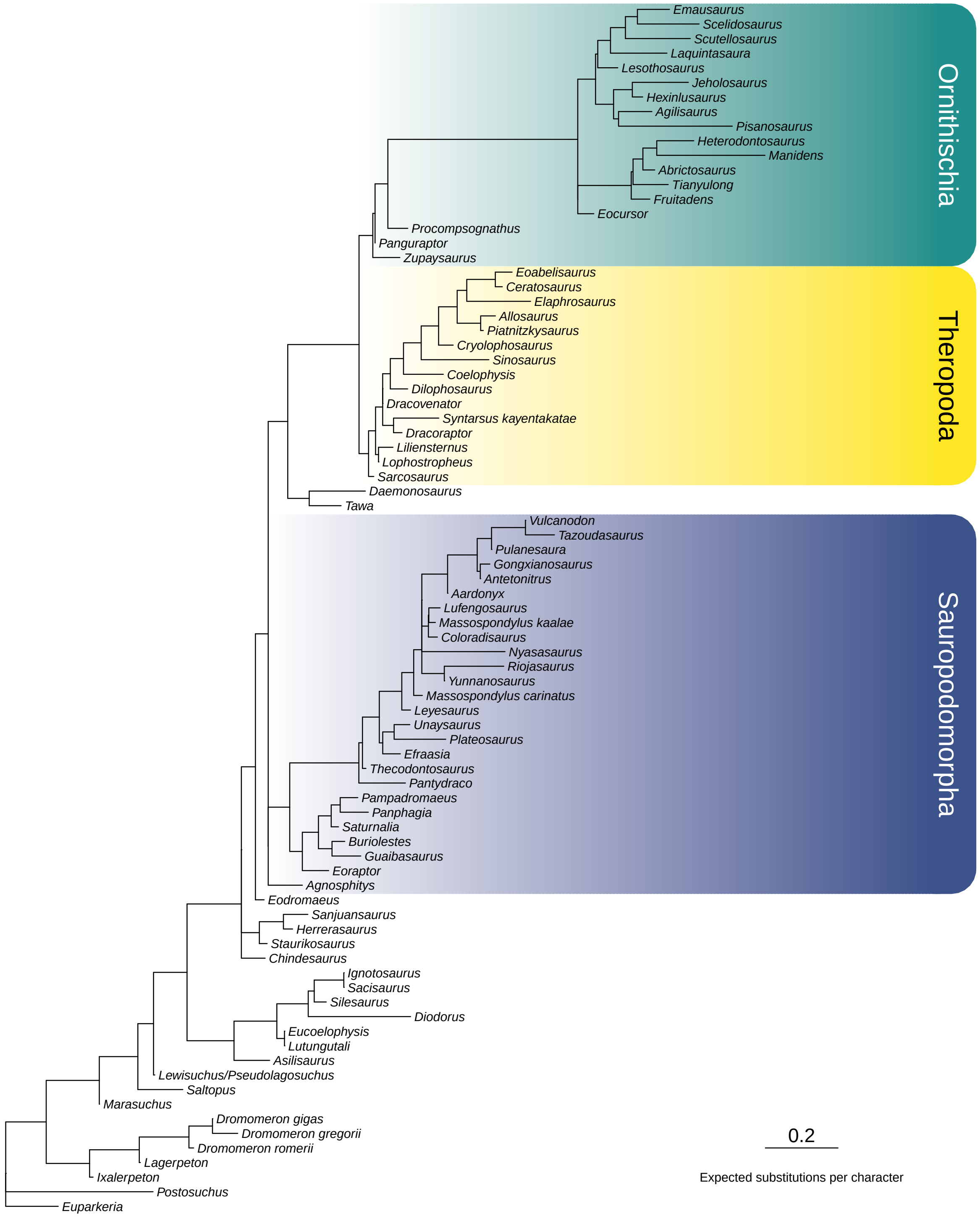

Supplementary Fig. 6: Maximum likelihood tree inferred for the original LEA matrix and constrained to recover Ornithoscelida ($\ln L = -7017.424$).

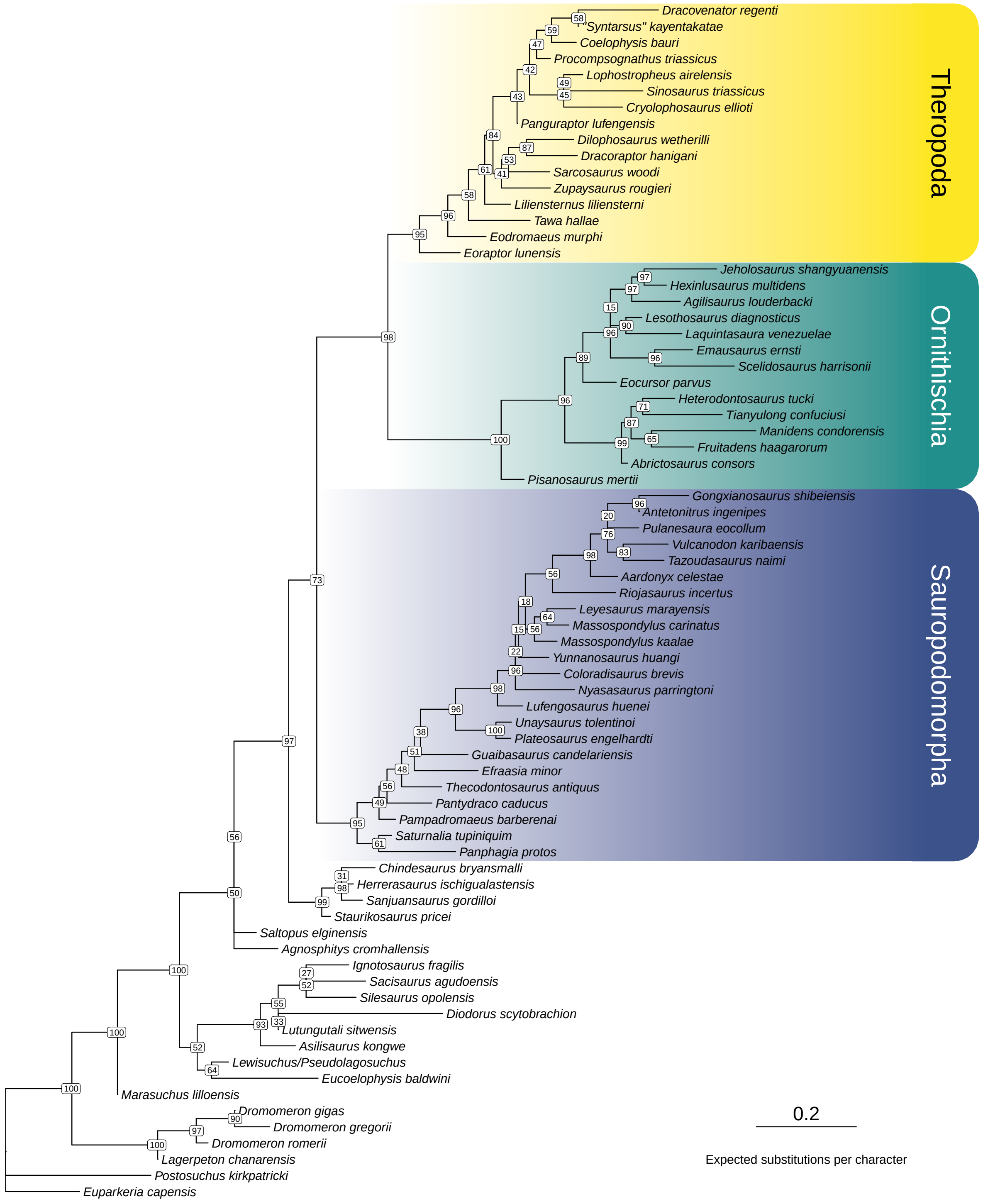

Supplementary Fig. 7: Maximum likelihood tree inferred for the BEA matrix without the single most decisive character (character 175; PS = 1.840) ($\ln L = -6353.365$). Node labels denote ultrafast bootstrap values computed from 1000 replicates.

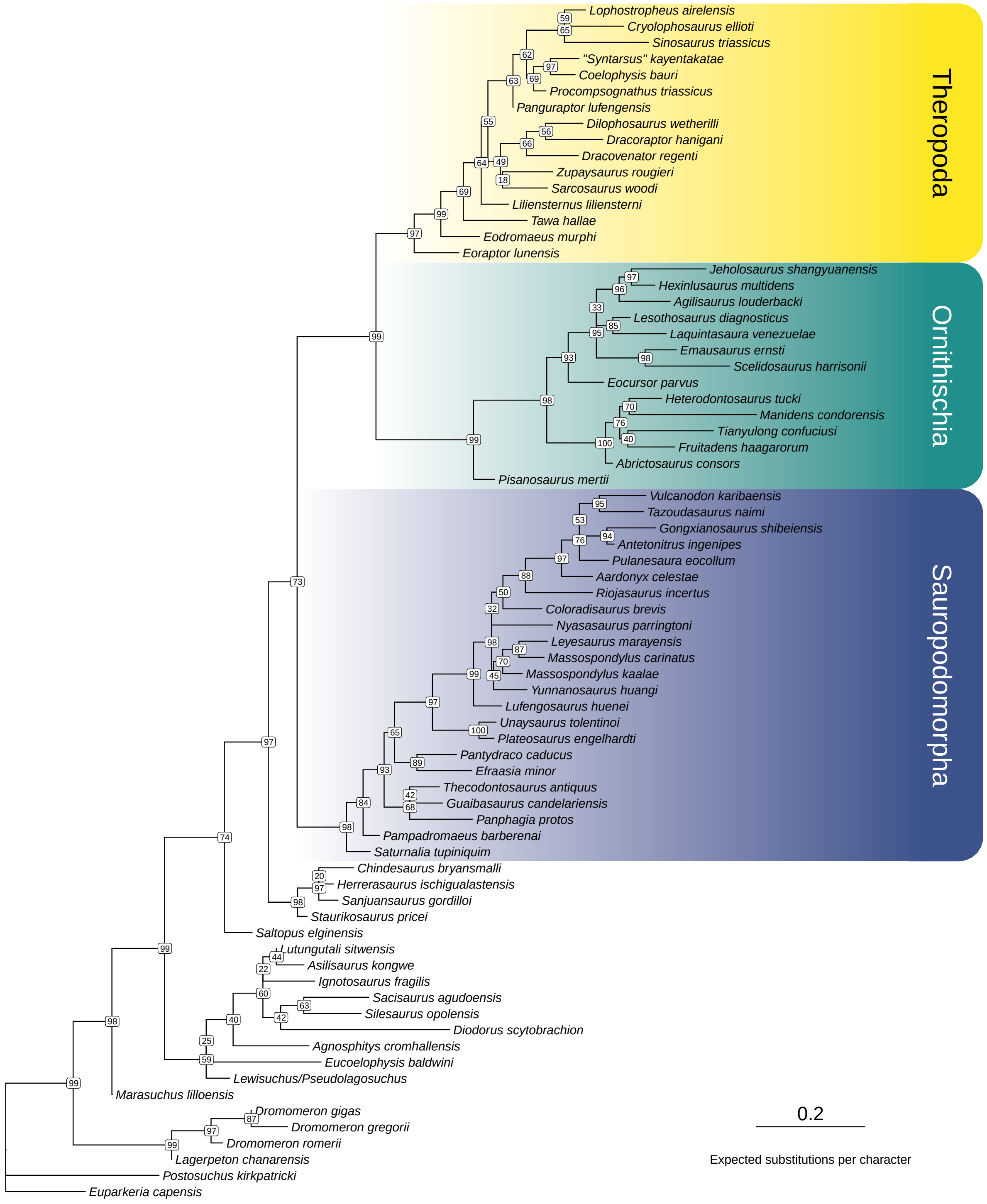

Supplementary Fig. 8: Maximum likelihood tree inferred for the BEA matrix without the 5 most decisive characters (characters 175, 174, 303, 37, 292; PS = 1.483–1.840) (ln $L = -6232.176$). Node labels denote ultrafast bootstrap values computed from 1000 replicates.

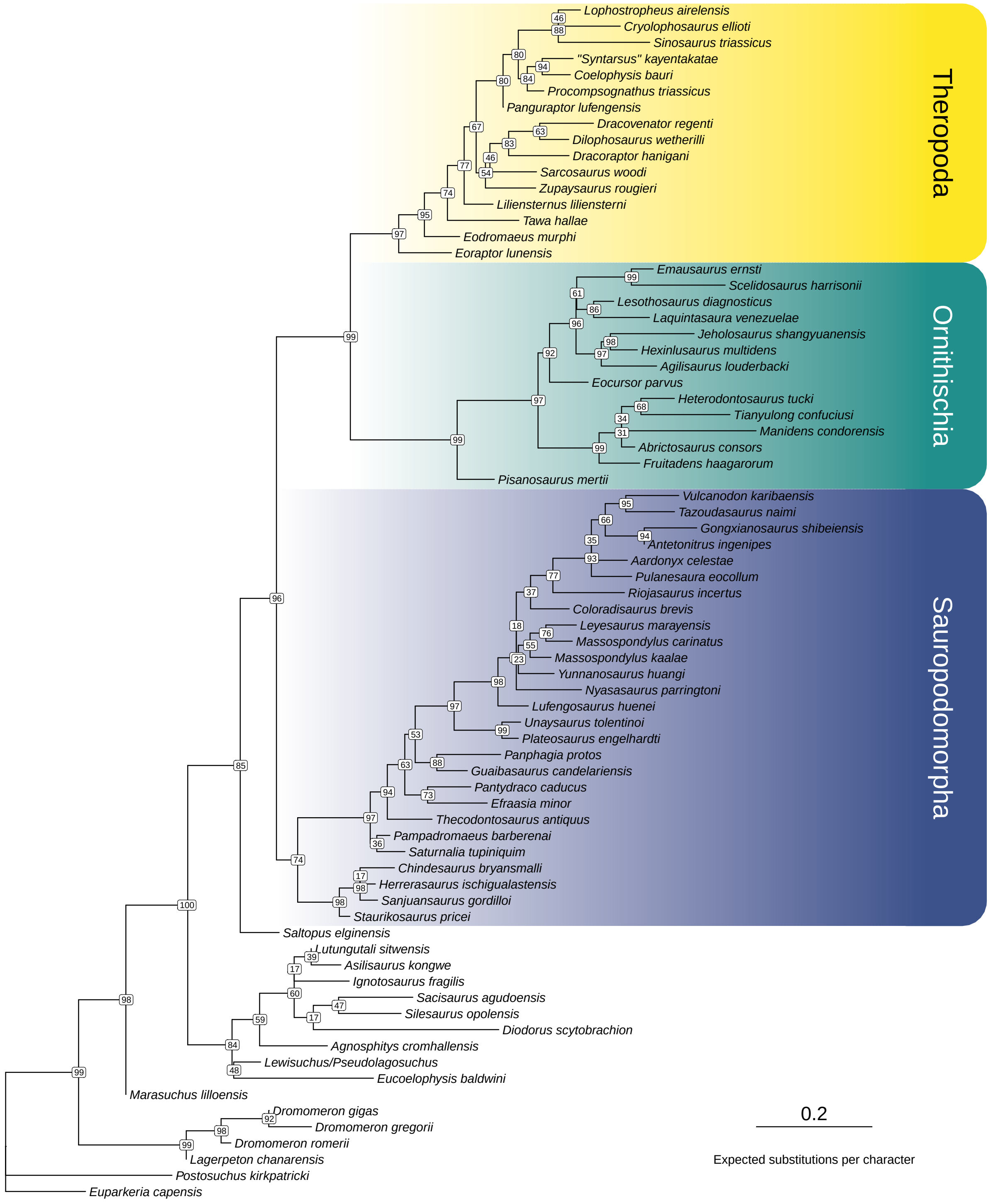

Supplementary Fig. 9: Maximum likelihood tree inferred for the BEA matrix without the 10 most decisive characters (characters 175, 174, 303, 37, 292, 353, 323, 387, 167, 411; PS = 1.382–1.840) ($\ln L = -6103.143$). Node labels denote ultrafast bootstrap values computed from 1000 replicates.

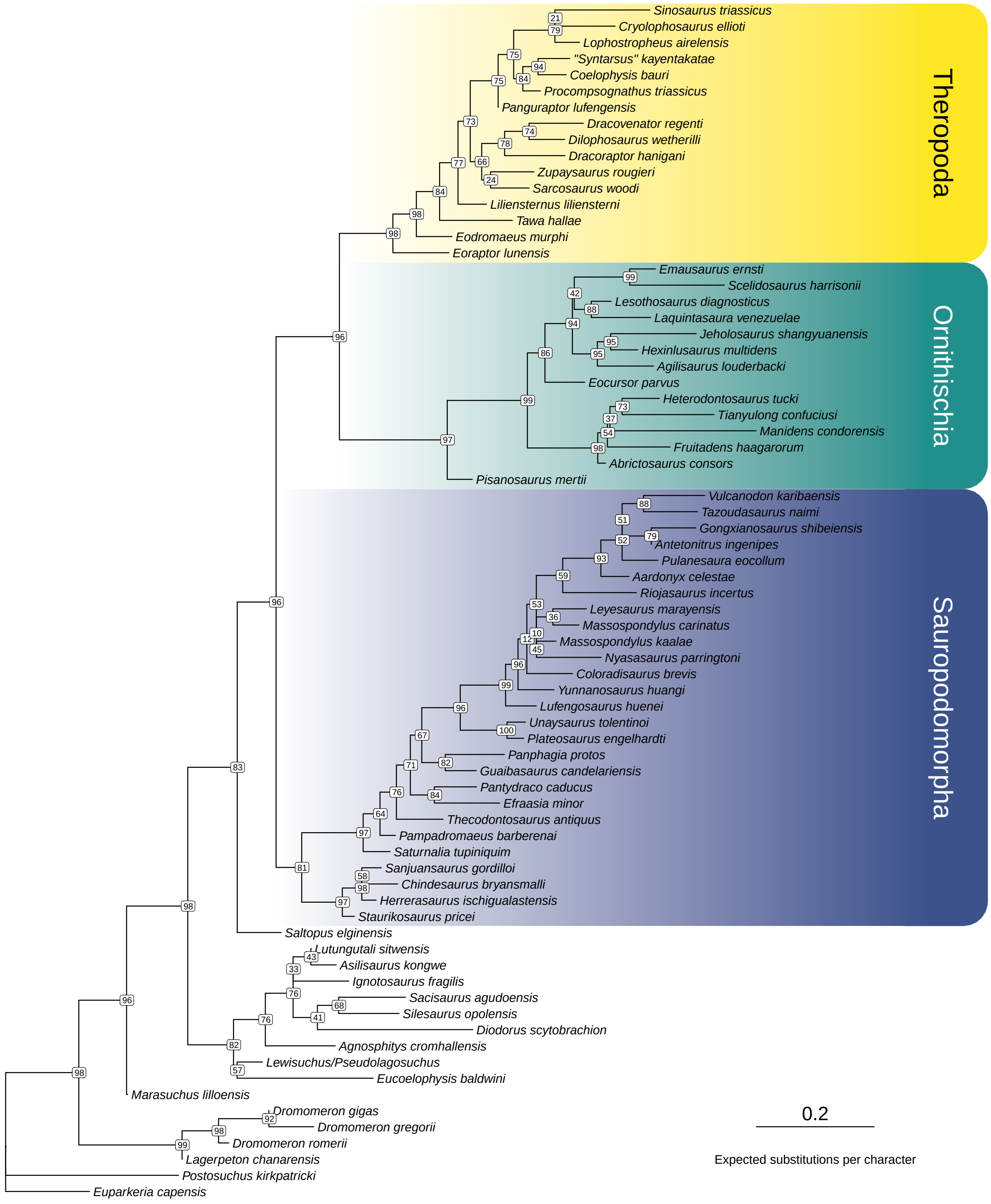

Supplementary Fig. 10: Maximum likelihood tree inferred for the BEA matrix without all characters with outlier PS values (characters 175, 174, 303, 37, 292, 353, 323, 387, 167, 411, 68, 360, 169, 444; PS = 1.337–1.840) (ln $L = -5992.320$). Node labels denote ultrafast bootstrap values computed from 1000 replicates.

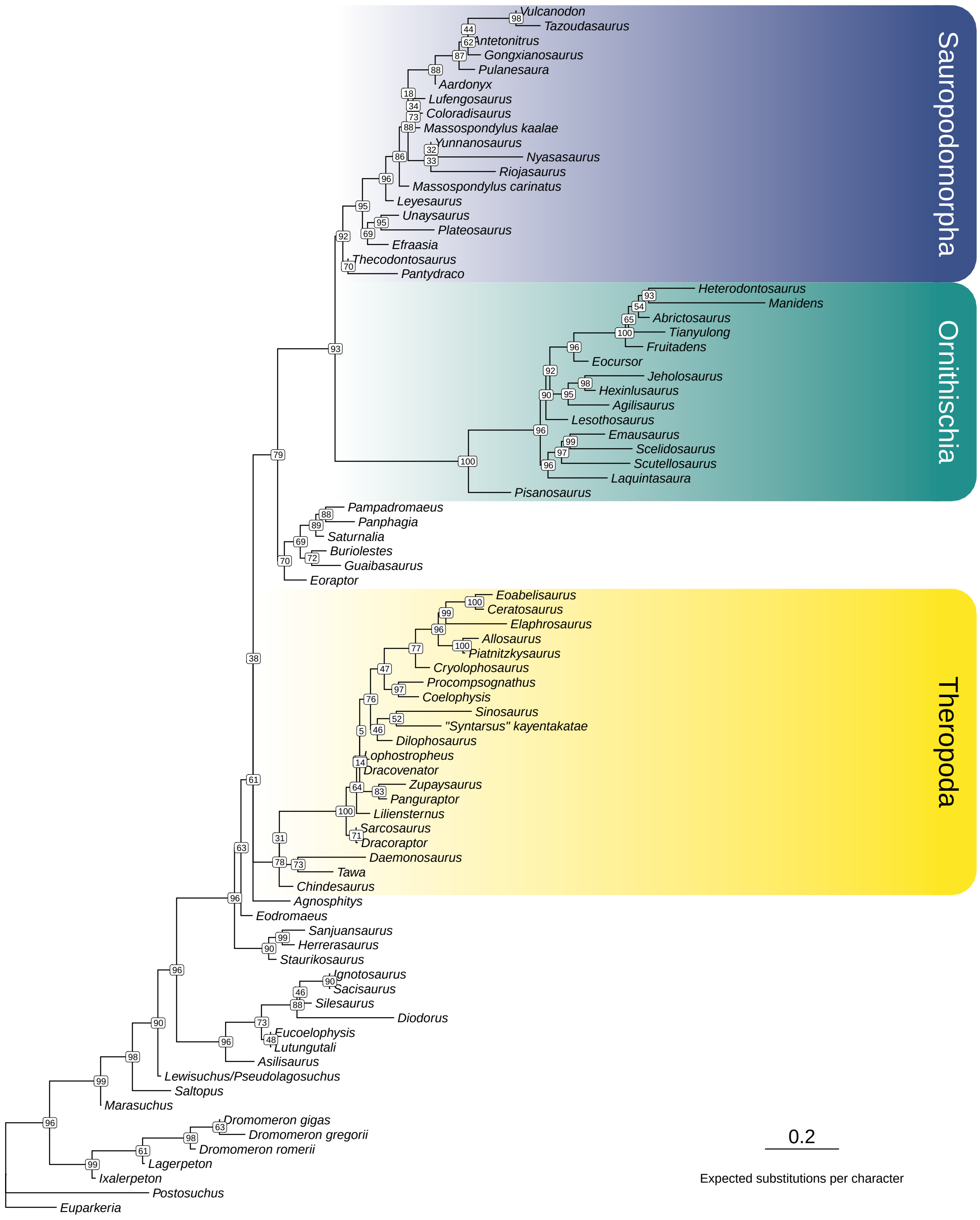

Supplementary Fig. 11: Maximum likelihood tree inferred for the LEA matrix without the single most decisive character (character 206; PS = 4.454) ($\ln L = -6972.146$). Node labels denote ultrafast bootstrap values computed from 1000 replicates.

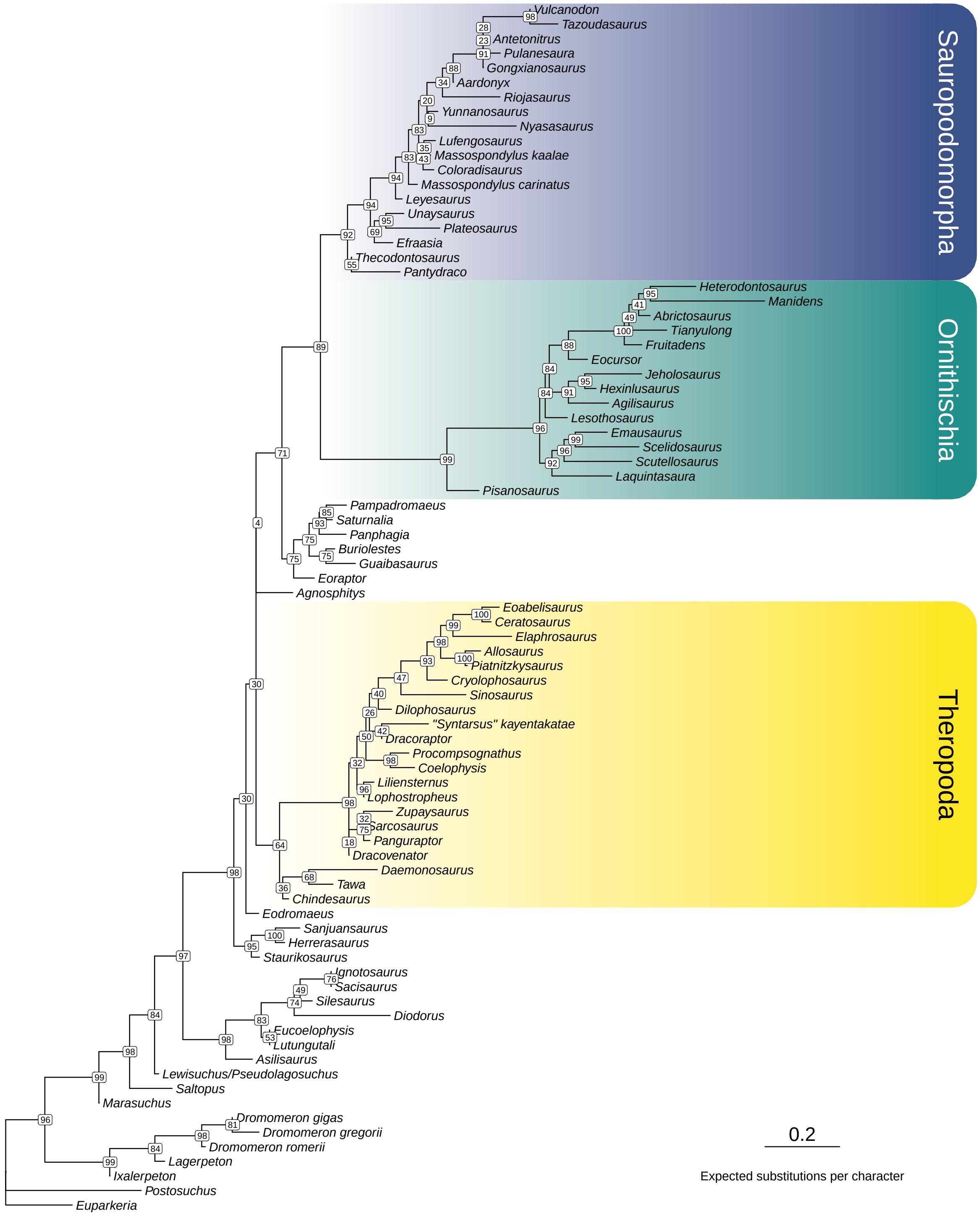

Supplementary Fig. 12: Maximum likelihood tree inferred for the LEA matrix without the 5 most decisive characters (characters 206, 318, 169, 391, 198; PS = 3.228–4.454) ($\ln L = -6869.597$). Node labels denote ultrafast bootstrap values computed from 1000 replicates.

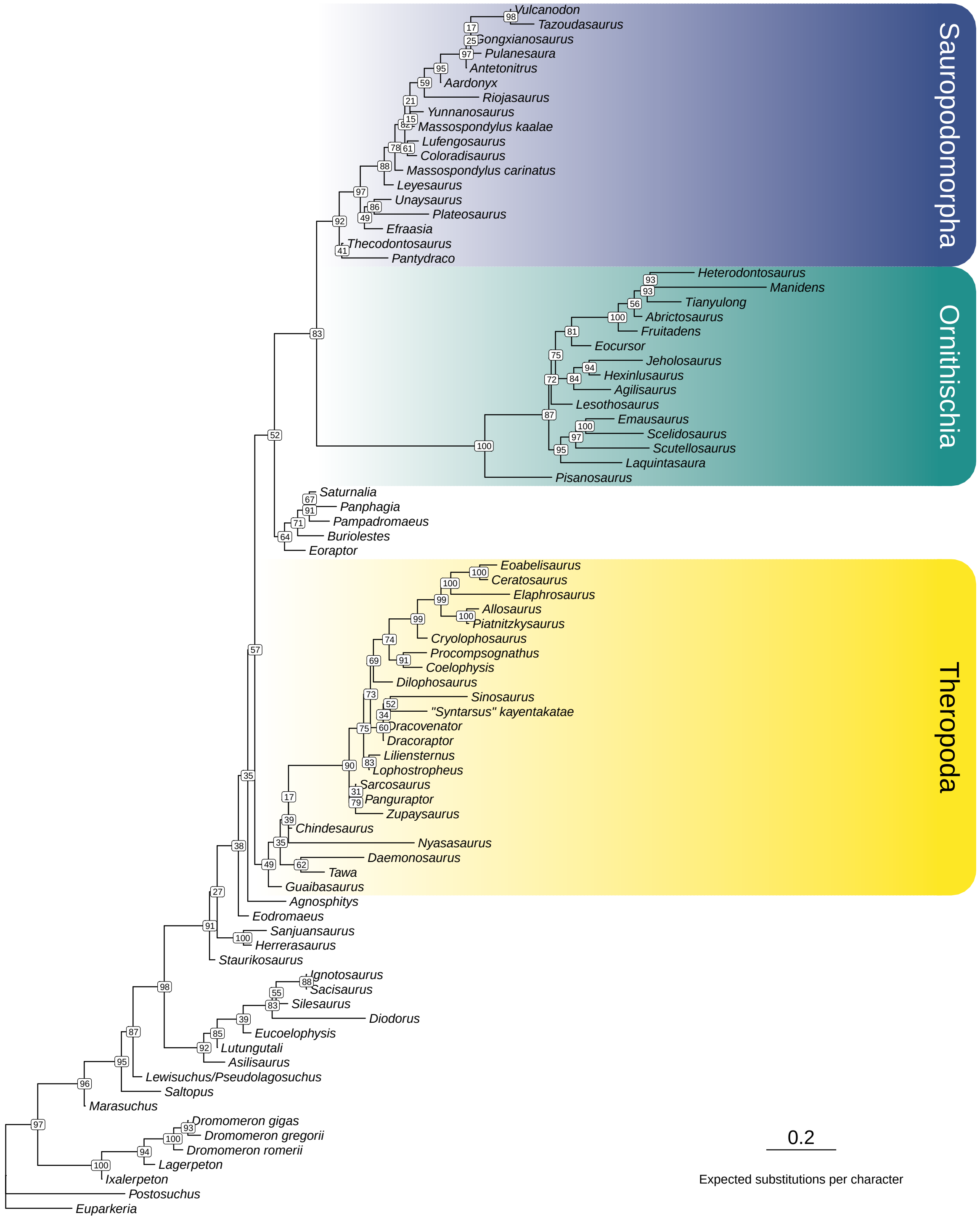

Supplementary Fig. 13: Maximum likelihood tree inferred for the LEA matrix without the 10 most decisive characters (characters 206, 318, 169, 391, 198, 338, 377, 306, 301, 367; PS = 2.311–4.454) ($\ln L = -6795.273$). Node labels denote ultrafast bootstrap values computed from 1000 replicates.

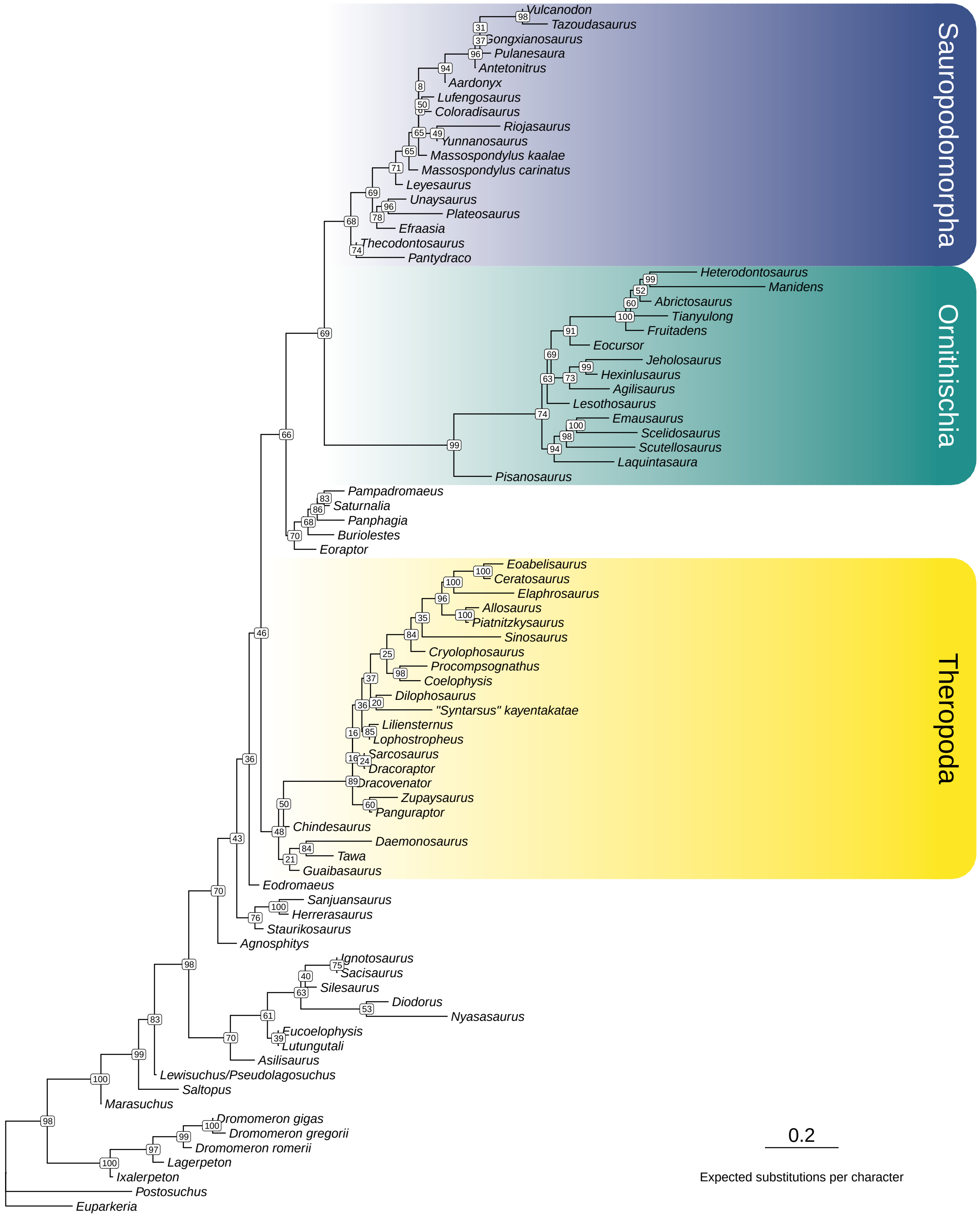

Supplementary Fig. 14: Maximum likelihood tree inferred for the LEA matrix without all characters with outlier PS values (characters 206, 318, 169, 391, 198, 338, 377, 306; PS = 2.716–4.454) (ln $L = -6825.859$). Node labels denote ultrafast bootstrap values computed from 1000 replicates.

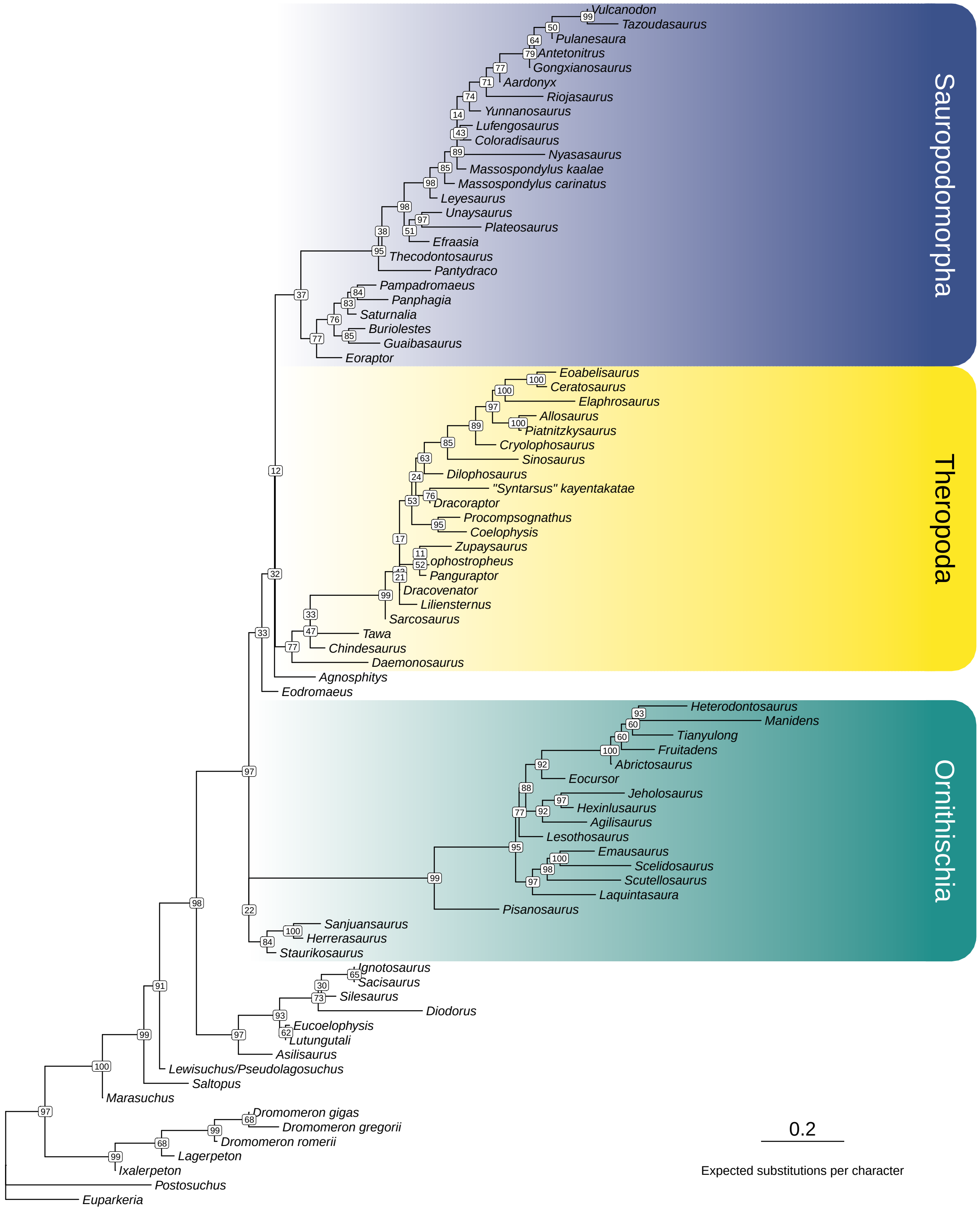

Supplementary Fig. 15: Maximum likelihood tree inferred for the LEA matrix with character 77 reverted to its original coding in the BEA matrix ($\ln L = -7013.107$). Node labels denote ultrafast bootstrap values computed from 1000 replicates.

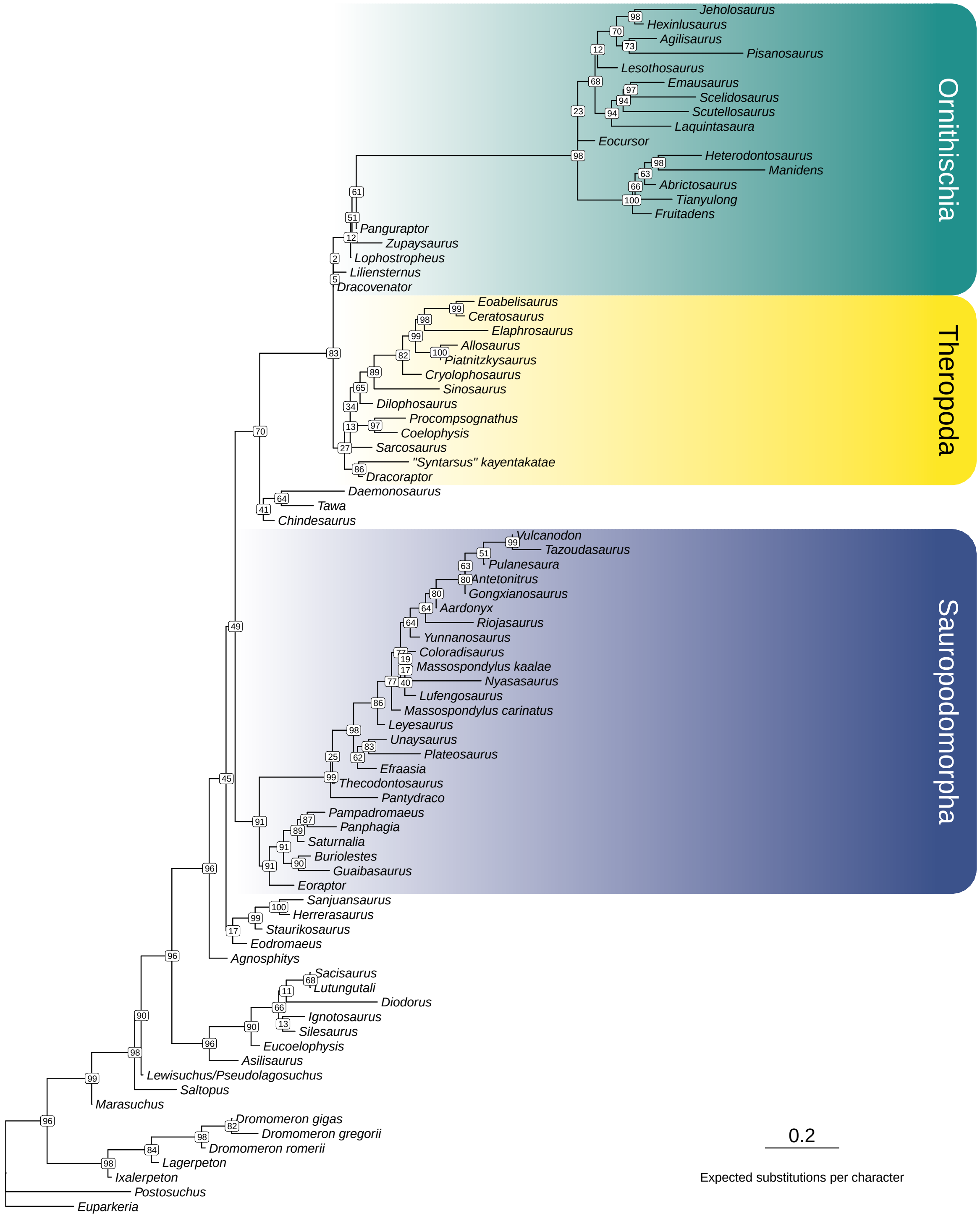

Supplementary Fig. 16: Maximum likelihood tree inferred for the LEA matrix with character 148 reverted to its original coding in the BEA matrix ($\ln L = -7010.895$). Node labels denote ultrafast bootstrap values computed from 1000 replicates.

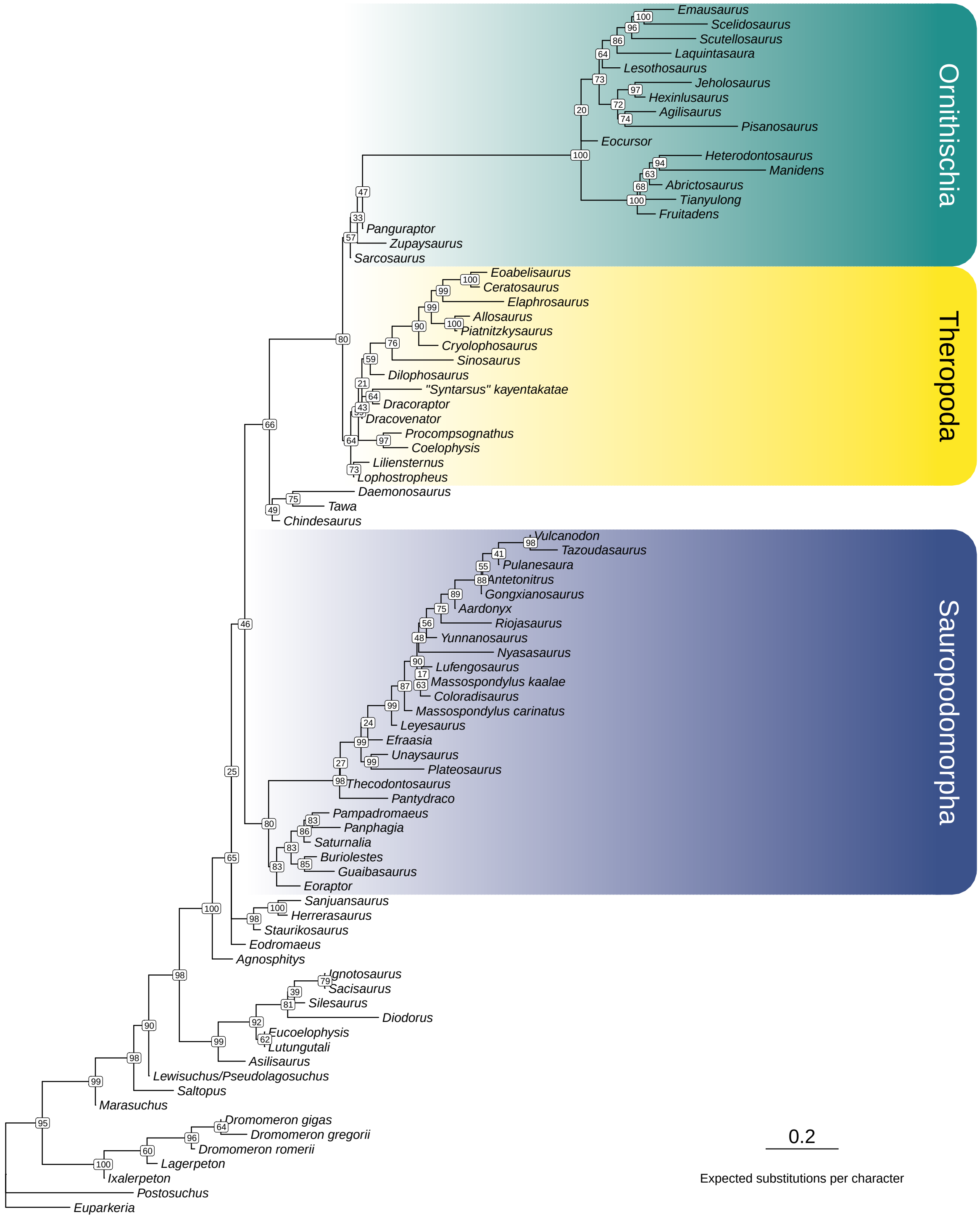

Supplementary Fig. 17: Maximum likelihood tree inferred for the LEA matrix with character 363 reverted to its original coding in the BEA matrix (ln $L = -7012.255$). Node labels denote ultrafast bootstrap values computed from 1000 replicates.

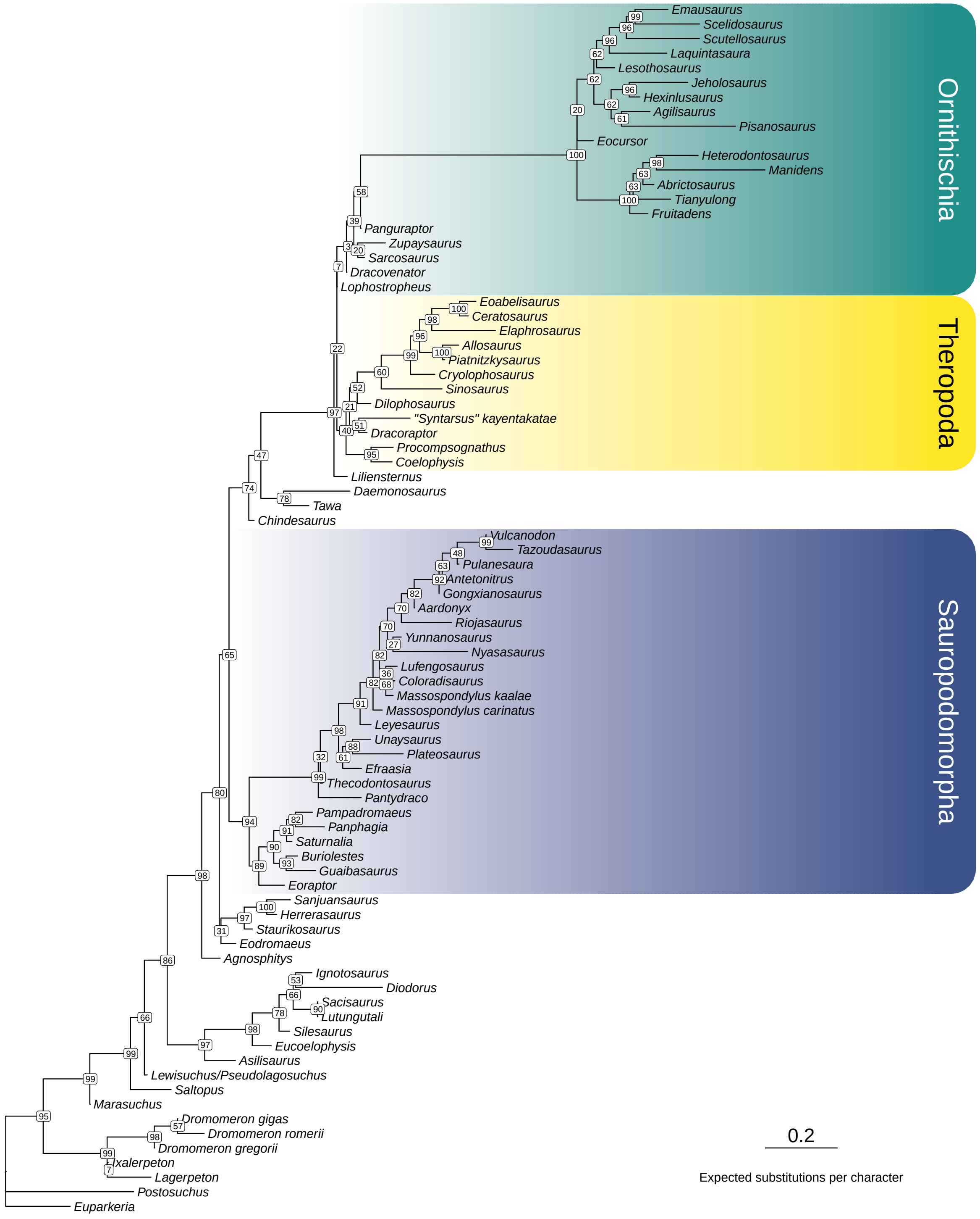

Supplementary Fig. 18: Maximum likelihood tree inferred for the LEA matrix with character 370 reverted to its original coding in the BEA matrix ($\ln L = -7023.812$). Node labels denote ultrafast bootstrap values computed from 1000 replicates.

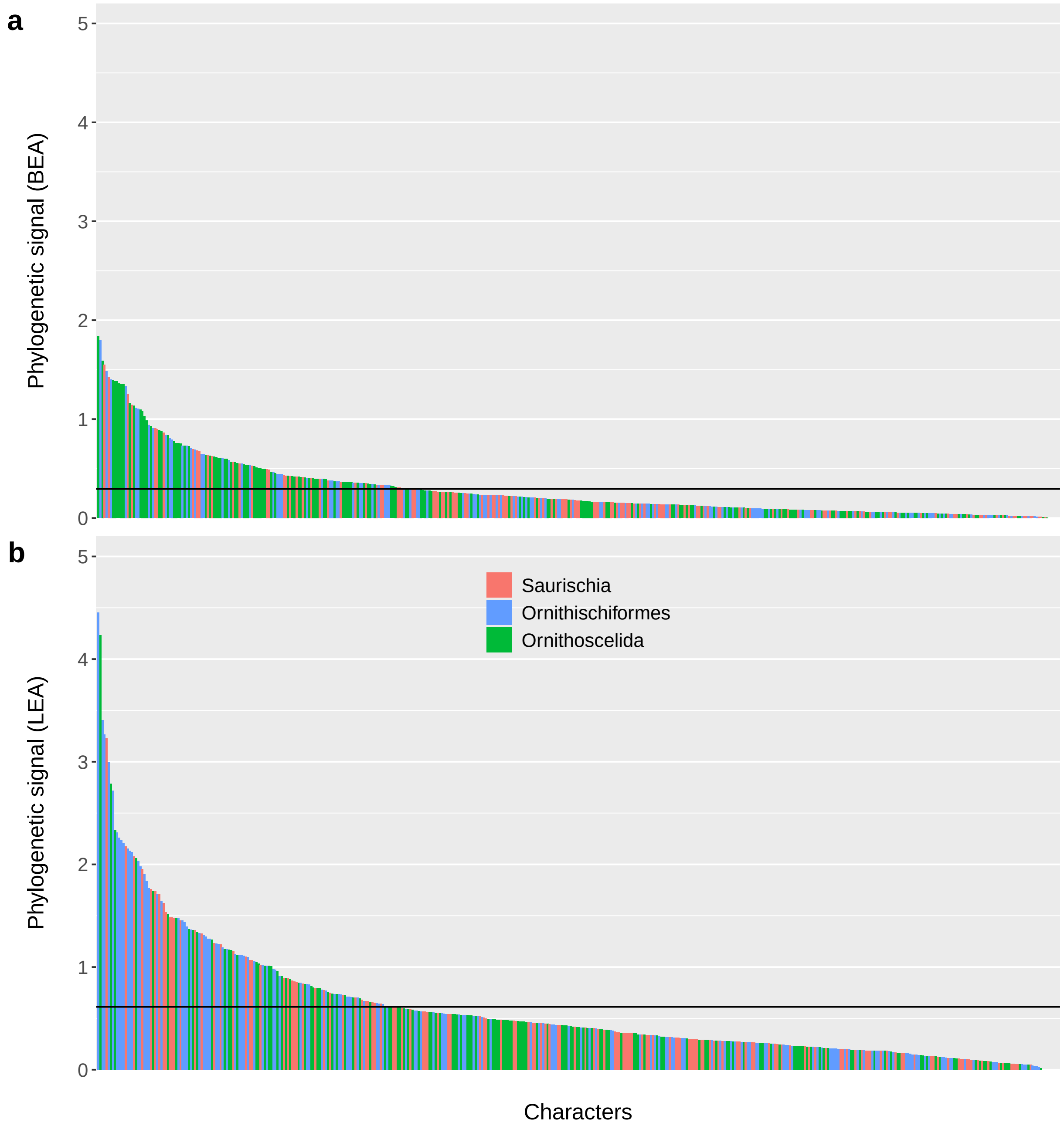

Supplementary Fig. 19: Distribution of phylogenetic signal (PS) across the (a) BEA and (b) LEA matrices. The figure corresponds to panels (a) and (c) of Fig. 2 in the main text, except that individual characters are arranged in descending order of their PS values and a common scale is imposed for both datasets. Additionally, the mean PS value in each dataset is denoted by a horizontal line.

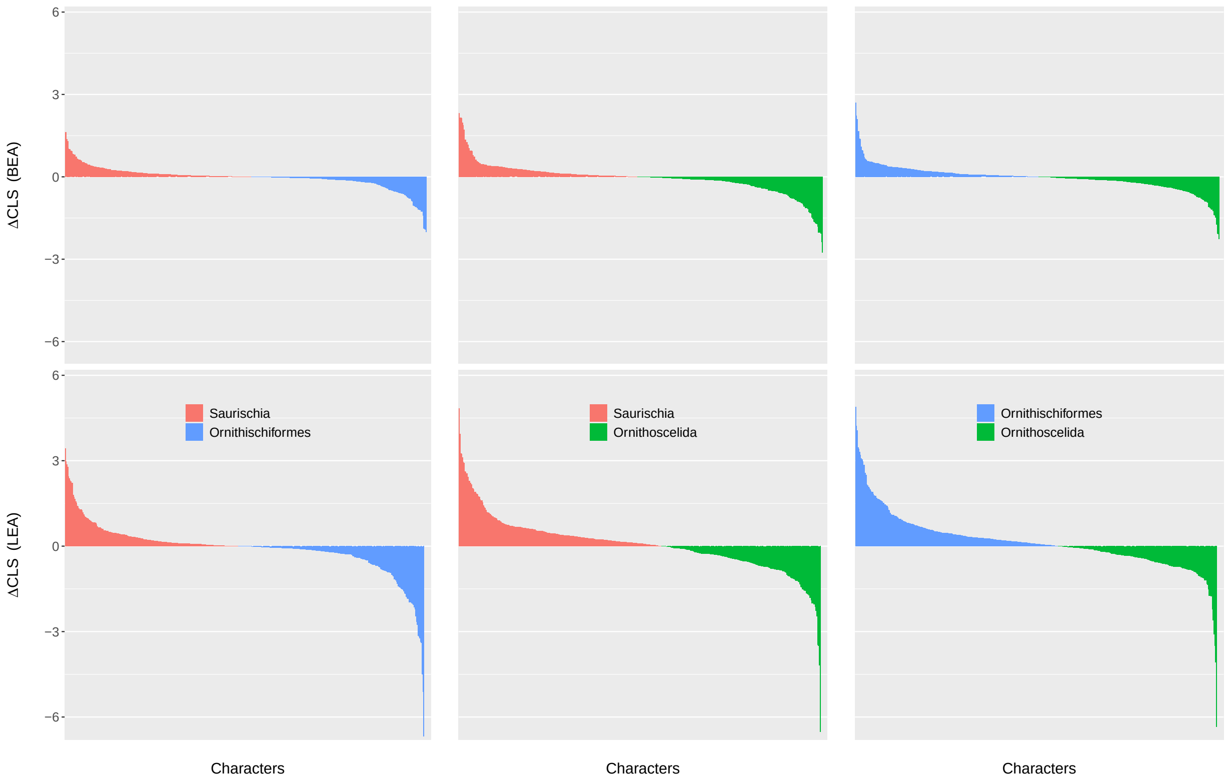

Supplementary Fig. 20: Pairwise comparisons of character support for the three alternative early dinosaur topologies. The figure corresponds to Fig. 3 in the main text, except that individual characters are arranged in descending order of their ΔCLS values and a common scale is imposed for both datasets.
